# The synergistic effect of waste cooking oil and endod (*Phytolacca dodecandra*) on the production of high-grade laundry soap

**DOI:** 10.1016/j.heliyon.2023.e16889

**Published:** 2023-06-01

**Authors:** Bahabelom Haile Abera, Abebe Diro, Tamene Tadesse Beyene

**Affiliations:** Department of Chemistry, College of Natural Sciences, Jimma University, P.O. Box 378, Jimma, Oromia, Ethiopia

**Keywords:** Laundry bar soap, Waste cooking oil, Endod (*Phytolacca dodecandra*), Saponification, Physicochemical properties

## Abstract

A green viewpoint based on the production of soap using waste products such waste cooking oils (WCOs) and Endod (*Phytolacca dodecandra*) is presented. The process of saponification, which involves reacting triglycerides with fats and oils in an alkaline solution, produces soap. With the help of WCO and Endod as manufacturing inputs, this study intends to create high-quality, commercially viable eco-friendly soaps. The optimal blend of WCO and Endod with sodium hydroxide solution was used in the current investigation to create laundry soaps. Evaluations were done on the cleansing effects and physico-chemical makeup of prepared soap. As a reference control, the raw oil soaps made without and with frying were employed. The free caustic alkali content, chloride content, moisture content, ethanol-insoluble-matter, total fatty matter, pH, and foam height values of the prepared soap were found to be in the range of 0%, 0%, 16.56–22.52%, 0.1–3.05%, 63.41–75.46%, 9.22–9.82%, and 3.3–8.1 cm respectively. The results obtained by blending fried WCOs and Endod were comparable to the Physico-chemical properties of the Endod-free uncooked/fresh oil soap. The soap made by blending WCO and Endod has higher cleansing power and better lather formation than the prepared soap with WCO without Endod. Moreover, the observed data are comparable with similar data reported in other literature, recommended acceptable standards (EAS, CES), and from many countries including the British, Malaysia, and the Philippines. Cooking oils fried at different temperatures do not have much effect on the quality of soap making. This suggested that the blending of WCOs and Endod can be used as raw materials to prepare high-quality and economically feasible soaps by replacing imported oils and fats.

## 1 Introduction

In the past, humans have proven an aggregate of animal fat and alkaline plant ash-made cleaning soap that lathered and wiped clean effectively [1]. Now a day, cleaning soap is ready via way of means of hydrolysis of animal fat and oils which might be dealt with alkali hydroxides like NaOH and KOH [2]. Soaps are cleaning agents (surfactants) [3] that contain discrete ends; a hydrocarbon give up this is hydrophobic and non-polar and may bind with oil or dirt, and a hydrophilic termination this is polar and soluble in water [4], used to smooth and wash pores and skin and clothing (Scheme S1).

Plant-supply oils and lengthy chain animal fat are used nearly in all cleaning soap-making processes [5,6]. The repetitive utilizations of vegetable oils and animal fat is predicted to result in the shortage of raw materials in soap industry. Moreover, it can additionally reason unwanted price increment in oil markets. Thus, continuous and sufficient supply may soon become difficult for the soap processing industries and also results in the supper expensive soaps that are frequently added to the society [7]. These facts forced researchers to search for alternative raw materials for soap making. Waste cooking oils (WCOs) could be a preferable candidate in this regard [8,9].

It is vital to observe that once oils are fried at temperatures of 180 °C and above for 5 or greater cycles, it creates a byproduct WCO [10,11]. Every day, a variety of establishments around the world generate this kind of oil, including restaurants, fast food outlets, households, and food processing industries [12]. Huge amounts of WCOs are discharged frequently into the environment without proper treatment [13,14]. Unfortunately, loads of those WCOs are improperly disposed of, which ends up in excessive environmental issues together with clogged sewage systems, enhanced water and waste dumping expenses, and detriment to aquatic existence because of the oil layer that hampers oxygen diffusion [15]. The disposal of WCO also causes economic loss [16,17]. Additionally, reusing deep-fried oil for cooking is detrimental to human health [18] and can lead to severe conditions like cancer, liver disease, and heart disease [[19], [20], [21]]. To prevent these problems, it is crucial to properly collect and recycle WCO to create value-added products like biodiesel, lubricants, biopolymers, and soap [[22], [23], [24]]. For example, using purified and bleached WCO to create expressly formulated soaps which can lower the free fatty acid/acid value and peroxide value while increasing the saponification value [[25], [26]]. In Ethiopia, many restaurants and street vendors overcook oils for several times and generate a significant amount of WCO due to a lack of awareness about the risks of overcooking. Therefore, using WCO as a raw material for industrial laundry soap production is vital.

The African soapberry plant *Phytolacca dodecandra*, known locally as Endod in Amharic, Handoodee in Afan-Oromo, and Shibti in Tigrigna, produces a range of saponins that have potent biological properties. These include anti-fungal, anti-bacterial, anti-protozoan, and anti-snails, which cause schistosomiasis, mosquito (Malaria) control, insecticidal, and spermicidal activities [27,28]. The plant’s fruits (berries) have been used for centuries in Africa as soap for washing clothes. When the small berries are dried, powdered, and placed in water, they yield a powerful foaming detergent solution. Endod also has a pleasant aroma dominated by aldehydes and ketones such as benzaldehyde, 2-nonanone, and sulcatone, and its essential oil contains major compounds like phytoene, phytol, and palmitic acid [29]. In Ethiopia’s favourable climatic conditions, the plant is a climber with drooping branches, grows very quickly, and bears fruit twice a year (Fig. S1) However, Endod has lost attention currently and is simply discarded because people prefer commercial soaps. Improving the Endod recovery rate, with the cooperation of citizens and effective collection programs, and changing it to a useful product like soap is fundamental. Therefore, by modernizing Endod, special attention should be paid to contributing to the economic growth of the country.

Thus, this study was initiated to use the optimized ratio of WCO and Endod for the production of qualified and cost-effective laundry soap by treating WCO and Endod samples with sodium hydroxide for the first time. Benefiting from the synergistic effect, the prepared soap qualified for the basic quality standards set for grade one laundry soap.

## 2 Materials and methods

### 2.1 Chemicals and reagents

Analytical standard chemicals such as carbon tetrachloride (99.8%, PubChem), Ethanol (96%, Merck), Potassium hydrogen phthalate (>99%, PubChem), potassium hydroxide (85%, Qualikems), sodium hydroxide (≥98%, Blulux Laboratories Ltd), hydrochloric acid (37%, Loba Chemie), phenolphthalein (PubChem), potassium iodide (99%, Alpha Chemika), Nitric acid (69%, Lobachemie), Silver nitrate (99.99%, Alpha Chemika), Acetone (99.99%, Fisher Scientific International Company), Potassium chromate, (99.99%, Fisher Scientific International Company) Light petroleum, Sulphuric acid (98%, Ankleshwar Gujarat, India), Sodium hydrogen carbonate (99%, TATA Chemicals), n-Hexane (99.0%, LobaChemie), methyl orange (Abronchemicals), Chloroform (99.8%, LobaChemie), analytical reagent quality, Glacial acetic acid (99.5%, Blulux laboratories), and Starch solution were used in this study. In addition, a standard food-grade edible vegetable cooking oil, WCOs, Endod berries powder, Fresh ginger, and double distilled water were also used for the study.

### 2.2 Material and apparatus

Several Materials/Apparatuses were used in this study. The moisture analyzer (Kern DAB 200-2 Weighing range (max.) 200 g, Readability 0.001 g), and pH meter (Seven Excellence pH/Ion meter S500-Std-K) were used during the investigation.

### 2.3 Sample preparation and pre-treatment

To investigate the influence of cooking on the physiochemical properties of pul oil, 10 L of a typical standard food-grade cooking oil was obtained and purposefully burnt at various temperatures over varying time durations. An aliquot of oil (1 L) was removed before to heating and labeled as unheated cooking oil (UHCO). The remaining 9 L oil is then poured into a stainless steel frying pan machine with a capacity of 10 L 1 kg of air dried potatoes were fed to the panning machine for each of the six frying sessions (Fig. S2a). A 1 L sample of this oil was taken and labeled as singly heated cooking oil (SHCO). The same processes were repeated to obtain oil heated 4 times to obtain repeatedly heated cooking oil (RHCO2-5) (Fig. S2b). We recorded the temperature and time consumed for each frying. The temperature and time consumed for each of the six frying sessions of RHCOs were 80 °C and 1 h, 140 °C and 1:45 h, 148 °C and 2:25 h, 182 °C and 3 h, 190 °C and 3:30 h, and 199 °C and 4:00 h, respectively. The process of heating and cooling the oil was performed without the addition of fresh oil. In addition, samples of viscous black WCOs were collected from hotels and restaurants that had undergone 80 frying sessions and 95 frying sessions respectively (Fig. S2c).

In order to remove solid inorganic particles from the collected WCOs, pretreatment procedures from the literature were utilized [9]. Fresh gingers were initially acquired from Jimma town (Fig. S3a). Fresh ginger roots were allowed to air dry for 2 h at room temperature after being peeled (Fig. S3b). The dried ginger roots were added to each 1 L sample of WCOs as a bleaching agent [52] as well as an antioxidant [[53], [54], [55]] to capture free radicals and poisonous substances released during the secondary oxidation stage of fatty acids. The pieces of dried ginger roots and WCOs were cooked together for 30 min at 90 °C while being gently stirred with a stirrer rod (Fig. S3c). The hot oil samples were then kept for some time to cool to room temperature. Then any existing impurities and contaminants were removed using suction filtration (Fig. S3d) and the filtered WCOs is kept for making soap (Fig. S3e and f).

Endod berries were collected from the trunk of the plant in the Jimma area to be used as filler and the active ingredient in the preparation (Fig. S4a). Impurities were removed from the seeds by gently washing them in fresh water while still attached to the stem (Fig. S4b). After that; the seeds were dried (Fig. S4c), berries and stems were separated (Fig. S4d and e), and crushed to powder using a grinder machine. The coarse and fine particles in the Endod berries powder were separated using a 90 μm analytical sieve (Fig. S4f). It is worth mentioning that only Endod powders below 90 μm particle size were used for the making of the appropriate soap.

### 2.4 Analysis of constituents of WCO

The two crucial parameters, Saponification Values (SV) and acid values (AV) were taken into consideration in this study to evaluate the qualities of the collected WCOs for recycling for soap making. To determine each parameter, the standard titration methods by the American Oil Chemists’ Society (AOCS) [56] and ISO 3657:2013, and ES ISO 660:2009 were used.

#### 2.4.1 Determination of saponification value

For the determination of the SV, 2 g of the oil sample was taken into a clean dried 250 mL of a conical flask. Then, 25 mL of 0.5 M KOH solution and some boiling chips were added and a reflux condenser was connected to the round-bottomed flasks. The flask was heated for an hour with frequent shaking. Later, 1 mL of 1% phenolphthalein indicator was added and the hot excess alkali was titrated with 0.5 mol/L of standardized hydrochloric acid solution until the pink color disappeared. At the same time and under the same circumstances, a blank titration had been conducted. Finally, the saponification value was calculated from the recorded data during titration using Equation (1).

(1) $$S.V=\frac{\left(V0-V1\right)\times C\times 56.1}{m}$$

where:

V_0_ is the volume in mL of the standardized HCl solution used for the blank test (22.5 mL), V_1_ is the volume in mL of the standardized HCl solution used for determination, C is the exact concentration of the standardized KOH solution used (0.5 N); m is the mass in g of the oil taken.

#### 2.4.2 Determination of acid value and acidity

To determine the AV, 10 g of oil sample were taken and added into the flask, 50 mL of ethanol was added into the second flask, and both were heated in a water bath at the same time at a temperature of 70 °C for 10 min. Next, 0.5 mL phenolphthalein was added to the 50 mL of heated ethanol. A neutralized ethanol was added to the test portion in the first flask, and it was mixed thoroughly. Then, a titration was made using 0.1 N of KOH solution until the definite pink color was formed, and the volume of the titrant was recorded at the endpoint.

The AV [57] and acidity or free fatty acid (AFFA) [58] content were calculated from the recorded data using Equation (2).

(2) $$AV=\frac{56.1\times N\times V}{m}$$

where

N is the exact concentration of standard KOH solution used in mole per litre, V is the volumetric standard KOH solution used in mL; m is the mass in g of the test portion, and M is the molar mass of the KOH (56.1 g/mol).

### 2.5 Preparation of laundry soap

During preparation, keeping the balance between oil and caustic/lye is necessary to maintain the quality of the prepared soap. If it is excessively oily, it is too soft and melts quickly, it will dry slowly and won’t froth well. An overly caustic substance will be unpleasant, dry, or burn your skin. No lye will be remaining in the soap bar if it is prepared properly. (3), (4), (5) were used in this study to balance the proper amount of oil, lye (NaOH), and water [59]. Dividing the SV value by 1402.50 or multiplying by the ratio of the molecular weights of NaOH/KOH (40/56.1), gives the amount of lye (NaOH) required for any unit, as long as you use the same units to measure your NaOH and Oils.

(3) $$Amount\;of\;lye\;in\;g=Amount\;of\;oil\;in\;g\times \left(\frac{S.\;V\;of\;the\;oil\times M.WNaOH}{M.WKOH}\right)$$

(4) (Amount of lye) ÷ 0.3 = (Total Weight of lye Water Solution in g)

(5) (Total Weight of lye Water Solution) − (Amount of lye) = (Amount of Water in g)

First, the oil’s SV was calculated based on Equation (1). After the careful calculation of SV, 100 g of the pretreated WCO 8 samples were heated to 60 °C and subsequently cooled to 40 °C in a separate flask. Next, 4% of Endod berries powder was blended in separate flasks, and then sodium hydroxide solution was added. The mixture was then agitated regularly using a rod stirrer for 15 min to create an intimate mixture. The same procedure was done for 1 sample of fresh oil (non-fried) without Endod. This is used as a reference standard control to determine the effect of temperature on the quality of soaps. To evaluate, the effects of Endod different Endod to oil ratio (0:100, 1:50, 1:33.33, 1:25, 1:20, 1:16.67) was used. In all samples after being poured into moulds, the soaps were allowed to air-dry for 24 h to harden.

### 2.6 Physicochemical tests of the prepared soaps

Literature indicated that the physicochemical characteristic of soap depends on several factors such physicochemical characteristics including FCA, Cl, pH, MC, EIM, TFM, and cleaning Power and lather formation [4,7,8]. The physicochemical properties of the soap samples were analyzed using ISO 456:1973, ISO 457:1983, ISO 672:1978, ISO 673:1981, ISO 685:1975, and standard procedures reported in the kinds of literature. The experiments were carried out in Triplicates.

#### 2.6.1 Determination of free caustic alkali (FCA)

For the determination of free caustic alkali, 5 g of soap was weighed followed by pouring 200 mL of ethanol into the flask. The flask was connected to the reflux condenser and boiled gently until the soap had completely dissolved. It was brought to a gentle boil and kept at the boil for 5 min to remove carbon dioxide. After it was cooled to about 70 °C, 4 drops of phenolphthalein indicator were added. Then, it was titrated with the ethanolic solution of hydrochloric acid (0.1 N) until the color is just perceptibly pink. The percentage of free caustic alkali in the soap expressed as sodium hydroxide Equation (6) is identical to ISO 456:1973:

(6) $$\%\;FCA=\frac{4\times V\times T}{m}$$

where

m is the mass in g of the test portion, V is the volume in mL of the ethanolic hydrochloric acid solution, and T is the normality of the ethanolic hydrochloric acid solution (0.1 N).

#### 2.6.2 Determination of chloride content (Cl)

For the determination of chloride content, 5 g of soap samples were dissolved in 50 mL of distilled water and then heated to dissolve the sample. The resulting solution was transferred into a 500 mL volumetric flask, the solution was stirred using a magnetic stirrer and heated at a temperature of 90–95 °C until the solution was completely dissolved. Next, the solution was titrated using HNO_3_ (1.4 N) until the pH reaches around neutrality. Finally, the resulting solution was titrated against 0.1 M AgNO_3_ using K_2_Cr_2_O_7_ as an indicator till a brick red color is obtained. The chloride content of the soap, expressed as a percentage by mass of sodium chloride (NaCl), is given by the formula Equation (7) [9]:

(7) $$\left(\%\right)Cl=\frac{V}{m}\times 0.585$$

where:

m is the mass in g of the test portion (5 g), V is the titrated volume in mL of the silver nitrate solution, and 58.5 is the mass in g of sodium chloride.

#### 2.6.3 Determination of moisture and volatile matter content (MC)

For the determination of the moisture and volatile matter contents, 5 g of samples were precisely weighed into a clean, dried dish. Then, it was heated in a moisture analyzer (Fig. S5) at a temperature of 105 °C until a constant mass and percentage result was observed. Then, necessary data was recorded from the moisture analyzer and were used for MC calculation based on Equation (8) [4].

(8) $$\%MC=\frac{m1-m2}{m1-m0}\times 100$$

where:

m_0_ is the mass in grams of the dish, m1 is the mass in g of the dish and the test portion before heating, and m_2_ is the mass in g of the dish and the test portion after heating.

#### 2.6.4 Determination of the content of ethanol–insoluble matter

Dissolution of the soap in ethanol, filtration, and weighing of the undissolved residue was done. The sample’s test portion, 5 g was put into a conical flask. Next, 200 mL of the ethanol was added to the test portion in the conical flask and connected to the reflux condenser. To avoid as much material sticking to the bottom of the flask as possible, it was heated to a slow boil while being swirled. Then, the filter paper was taken out of the desiccator after 1 h and left in there just long enough for it to cool entirely to room temperature and weighed it.

The content of EIM expressed as a percentage by mass is given by Equation (9) identical to ISO 673:1981.

(9) $$\left(\%\right)EIM=\frac{m}{mo}\times 100$$

where:

m_0_ is the mass in g of the test portion, and m is the mass in g of the residue (mass of dish and mass of filter paper with final residue – the mass of dish).

#### 2.6.5 Determination of total fatty matter content (TFM)

To determine the total fatty matter content of the prepared sample, 5 g of the test samples were added into the beaker. The test portion was dissolved in 100 mL of hot water. The solution was poured into the separating funnels. The beaker was washed with small quantities of water and added the washings to the separating funnel. Then, a few drops of methyl orange solution were added as an indicator, and while violently shaking the separating funnel, a precisely measured known volume of hydrochloric acid solution (1 N) was added until the color of the solution turned pink, indicating that there was an excess of around 5 mL. 100 mL of the light petroleum was then added after the separated funnel’s contents had been cooled to roughly 25 °C. The stopper was inserted and gently inverted the separating funnel whilst maintaining a hold on the stopper. The stop-cock of the separating funnel was opened gradually to release any pressure, then close, gently shaken, and again release the pressure. The shaking was repeated until the aqueous layer had become clear, and then allowed to stand for 5 min. After that, the extracted colorless solution (a mixture of petroleum and oil) was collected in the conical flask and transferred to the pink color solution. The residue was dissolved in 20 mL of the ethanol, a few drops of the phenolphthalein solution was added, and then the ethanolic potassium hydroxide solution (1 N) was titrated to a faint permanent pink color, and the result was recorded. Next to that, the ethanol solution was evaporated into the water bath. When the evaporation was near completion, the flask was rotated to distribute the potassium soap in a thin layer on the sides and bottom of the vessel. The preliminary drying of the potassium soap was Carried out a by adding acetone and evaporating off the acetone on the water bath under a slow stream of cold dry nitrogen or air. Then, it was heated to constant mass in the oven, controlled at 105 ± 2 °C, i.e. until the difference in mass after heating for an additional 15 min does not exceed 3 mg. It was ultimately weighed after cooling in a desiccator.

The total fatty matter content is given, as a percentage by mass, by Equation (10) identical to ISO 685:1975:

(10) $$\%TFM=\left[m_{1}-\left(V\times T\times 0.038\right)\right]\times \frac{100}{mo}$$

where:

m_1_ is the mass difference in g between the final flask and initial flask (mass of fat matter), m_o_ is the mass in g of the test portion, V is the volume in mL of the stand and volumetric ethanol potassium hydroxide solution used for the neutralization, T is the exact normality of the standard volumetric ethanolic potassium hydroxide solution.

#### 2.6.6 Determination of pH of aqueous solutions

To measure the pH of the aqueous solution, 99 g of distilled water and 1 g of soap sample were weighed out, and the water was heated to 70 °C. Then, soap was added to the distilled water and thoroughly mixed with a magnetic stirrer until the soap was completely dissolved. The pH of the solution was then determined using a pH meter after being chilled in an ice bath at 40 °C [4].

#### 2.6.7 Test of cleaning power, foam formation, stability, and foam removal by the water of the prepared laundry soap

**Fig. 1:**
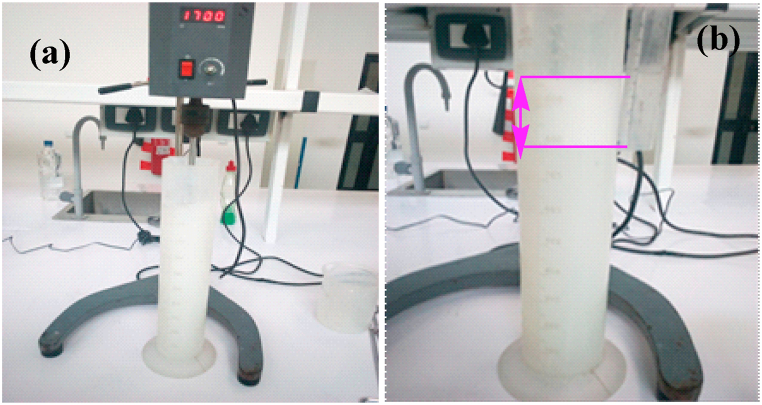
Foam Formation and Stability of the made laundry soap a) Stirred for 10 min to generate foam b) Measuring the height of the foam.

The cleaning capacity of the made soap bars was evaluated using a white, extremely dirty cloth that had been dumped on the road and was highly smeared with oil. The capacity of soap to foam allows it to produce lather when used [60]. Its foam formation and stability were evaluated by adding 4 g of soap and 400 mL of tap water into an 800 mL measuring cylinder [6]. The mixture was continuously stirred with a magnetic stirrer (Fig. 1a) to generate foams. After continuing stirring at 1700 rpm for 5 min, the mixture was allowed to stand still for 2 min to measure the generated foam and allowed to stand still for 10 min to measure the stability of the foam. The foam height of the solution generated and stabilized within the sample was measured and recorded (Fig. 1b).

## 3 Results and discussions

### 3.1 Analysis of physico-chemical properties of the oils

In this study, SV and AV were investigated to determine the physicochemical properties of the oils, and the experimental results are shown in (Table 1).

**Table 1:** The experimental results of the SV and AV of the oils.

Types of the oils	Temp. in (°C)	Time	Parameters	
			SVof the oils	AV of the oils
Not-frying/fresh oil	–	–	252.450 ± 0.000	0.620 ± 0.000
Frying cycle 1	80	1:00 h	248.170 ± 0.070	0.700 ± 0.030
Frying cycle 2	140	1:45 h	245.440 ± 0.000	1.190 ± 0.010
Frying cycle 3	148	2:25 h	241.930 ± 0.700	1.770 ± 0.030
Frying cycle 4	182	3:00 h	239.830 ± 0.000	2.380 ± 0.020
Frying cycle 5	185	3:30 h	236.320 ± 0.700	2.890 ± 0.030
Frying cycle 6	199	4:00 h	231.410 ± 0.000	4.070 ± 0.030
WCO collected from hotel 80 frying cycle	220	5 days	197.050 ± 0.700	4.410 ± 0.030
WCO collected from restaurant 95 frying cycle	250	7 days	194.25 ± 0.700	4.520 ± 0.030

Different studies demonistrated that repeatedly heating vegetable oil at high temperatures could cause oxidation, which results in colour change, rancid odour, and rancid flavour [18,61]. WCOs need to be pretreated (purified and bleached) before being reused to improve their quality [26] and finally analyzed to check their suitability for soap production [9]. For this reason, purification and bleaching of the WCO were done to improve their quality, and their suitability for soap production was assessed using the parameters such as SV and AV.

SV reveals the average molecular weight and, consequently, chain length of the fatty acids in the oil or fat [6]. SV is reasonable means of characterizing oil, especially for soap production [61]. The SV (mg KOH/g oil) denotes the presence of normal triglycerides, which require a higher concentration of alkali to complete the saponification reaction [62]. The SVs of different samples involved in this investigation are given in Table 1. Accordingly, the SV for frying, frying 1, frying 2, frying 3, frying 4, frying 5, frying 6, and WCO collected from hotels and restaurants frying 80, and 95 were, 252.45 ± 0.000, 248.17 ± 0.070, 245.44 ± 0.000, 241.93 ± 0.700, 239.83 ± 0.000, 236.32 ± 0.700, 231.41 ± 0.000, 197.05 ± 0.700, and 194.25 ± 0.700 mg KOH/g respectively. The result indicated that SV decreased with the frying cycle. It is worth mentioning that the SV of WCO collected from hotel values (194.25 ± 0.700 mg KOH/g) and a restaurant (252.45 ± 0.000 mg KOH/g) compared to the laboratory-treated samples is the least. This is due to the effect of overheating which results in the decomposition of the oil as was also indicated in several pieces of literature [64]. The more the SVs, the better capable the oil or fat is of manufacturing soap. The SVs ranged from 126.8 mg KOH/g to 221 mg KOH/g [46], and according to NAFDAC and ISO 3657:2013 as well as other research papers’ ranged from 192 mg KOH/g to 250 mg KOH/g [2,34] suggested well in soap production. Thus, all of the WCOs utilized in the study were shown to have comparable SV and are appropriate for soap production.

The AV, which results from enzymatic activity, indicates the quality of the fatty acids in the oil. Oil with a low AV is long-lasting and resistant to peroxidation and rancidity. The SV of oil is used to assess if it is safe to eat and appropriate to use in the soap industry. Oil samples with higher free fatty acid contents are found to have a higher AV, which donates decreased oil quality [65]. The AV of samples of WCO involved in the current investigations are given in Table 1. From the table, we can see that the respective AV of the fresh oil, frying cycle 1, frying cycle 2, frying cycle 3, frying cycle 5, frying cycle 6, hotel frying cycle 80, and restaurant frying cycle 95 are 0.62 ± 0.000, 0.70 ± 0.030, 1.19 ± 0.010, 1.77 ± 0.030, 2.38 ± 0.020, 2.89 ± 0.03, 4.07 ± 0.030, 4.41 ± 0.03, 4.52 ± 0.0 mgKOH/g. The results from the study indicated that as the oil is overheated or the frying cycle increases, the AV of the oil also increases. Consequently, the quality of fatty acid decreases, which causes oxidation, rancidity, and deterioration of the oil [45]. Literature indicates that oils with AV less than 4 mg KOH/g are safe for consumption [1,46]. Moreover, studies indicated that oils having higher AV (up to 16.1 mgKOH/g) [1,47], are recommended for good-quality soap preparation. As a result, it can be inferred from this study that oils labeled as frying 1 to (heated from 80 °C to 185 °C) met the requirements of the food domain as their AV values were relatively lower (<4.00 mg KOH/g) [57] while oils used for frying 6, a hotel-80 and a restaurant-95 having AV above 4 mg KOH/g are recommended for the production of soaps. Thus the result is this study indicated that AV of WCO collected hotels and restaurants fall in the recommended range for soap production.

### 3.2 The prepared laundry bar soaps

**Fig. 2:**
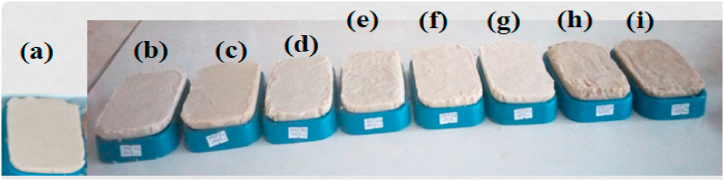
The prepared laundry bar soap Endod to oil ratio (1:25, W/W), except “a”. (a) soap from non-fried oil without Endod (b) soap from Frying 1 (c) soap from Frying 2 (d) soap from Frying 3 (e) soap from Frying 4 (f) soap from Frying 5 (g) soap from Frying 6 (h) soap from WCO collected from the hotel frying-80 (i) soap from WCO collected from the restaurant -95.

To determine the effects of over-cooking oil at different temperatures on the quality of the soaps, 6 soaps from frying 1 to 6, and 2 soaps from frying-80 and frying-95 were prepared (Fig. 2a–i). The results of soap prepared from pre-treated oil (Fig. 2b–g) or collected waste oil (Fig. 2h–i) were compared with soap made from non-fried oil without Endod which is used as a reference control (Fig. 2a). The physicochemical properties of the prepared soaps were comparable with other works reported in the works of literature [1,45,57].

**Fig. 3:**
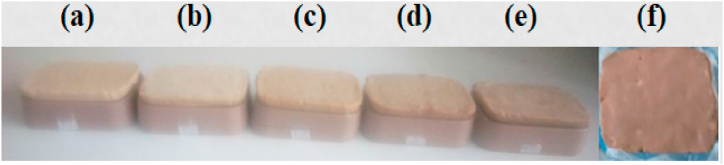
Soap bars prepared from the Frying 6 WCO (Fig. 2f) and in different Endod to oil ratios a) 1:16.67, b) 1:20, c) 1:25, d) 1:33.33, e) 1:50, f) 0:100 (without Endod).

To increase the cleaning action of the prepared soap, the pretreated and crushed Endod berries were mixed frying 6 based on the result from Table 1. Accordingly, the effects of varying Endod ratios in the fixed amount of WCO were also examined (Fig. 3a–f). The result indicating the effects of varying amounts of Endod in a fixed amount of WCO were given for the different parameters in the follow-up sections (Table 2, Table 3, Table 4, Table 5, Table 6, Table 7, Table 8 under section ‘b’).

**Table 2:** Experimental results FCA of the prepared laundry bar soaps.

Soap products prepared from	The blended ratio of Endod and oil (W/W, E/O)	FCA (%)
**Constant ratio of Endod and oil in different frying cycles (a)**		
Not-frying/fresh	0:100	0.00 ± 0.000
Frying cycle 1	1:25	0.00 ± 0.000
Frying cycle 2	1:25	0.00 ± 0.000
Frying cycle 3	1:25	0.00 ± 0.000
Frying cycle 4	1:25	0.00 ± 0.000
Frying cycle 5	1:25	0.00 ± 0.000
Frying cycle 6	1:25	0.00 ± 0.000
WCO collected from hotel 80 frying cycle	1:25	0.00 ± 0.000
WCO collected from restaurant 95 frying cycle	1:25	0.00 ± 0.000
**Different ratio of Endod and the same amount of oil in the same frying cycle (b)**		
Frying cycle 6	0:100	0.00 ± 0.000
Frying cycle 6	1:50	0.00 ± 0.000
Frying cycle 6	1:33	0.00 ± 0.000
Frying cycle 6	1:25	0.00 ± 0.000
Frying cycle 6	1:20	0.00 ± 0.000
Frying cycle 6	1:17	0.00 ± 0.000

**Table 3:** Experimental results of Chloride content of the prepared laundry bar soaps.

Soap products prepared from	The blended ratio of Endod and oil (W/W, E/O)	%Cl^−^
**Constant ratio of Endod and oil in different frying cycles (a)**		
Fresh	0:100	0.00 ± 0.000
Frying cycle 1	1:25	0.00 ± 0.000
Frying cycle 2	1:25	0.00 ± 0.000
Frying cycle 3	1:25	0.00 ± 0.000
Frying cycle 4	1:25	0.00 ± 0.000
Frying cycle 5	1:25	0.00 ± 0.000
Frying cycle 6	1:25	0.00 ± 0.000
WCO collected from hotel 80 frying cycle	1:25	0.00 ± 0.000
WCO collected from restaurant 95 frying cycle	1:25	0.00 ± 0.000
**Different ratio of Endod and the same amount of oil in the same frying cycle (b)**		
Frying cycle 6	0:100	0.00 ± 0.000
Frying cycle 6	1:50	0.00 ± 0.000
Frying cycle 6	1:33	0.00 ± 0.000
Frying cycle 6	1:25	0.00 ± 0.000
Frying cycle 6	1:20	0.00 ± 0.000
Frying cycle 6	1:17	0.0 ± 0.0000

**Table 4:** The experimental results of moisture contents of the prepared laundry bar soap.

Soap products prepared from	The blended ratio of Endod and oil (W/W, E/O)	%MC
**Constant ratio of Endod and oil in different frying cycles (a)**		
Not-frying/fresh	0:100	20.07 ± 0.020
Frying cycle 1	1:25	16.41 ± 0.050
Frying cycle 2	1:25	17.17 ± 0.050
Frying cycle 3	1:25	17.54 ± 0.010
Frying cycle 4	1:25	18.15 ± 0.010
Frying cycle 5	1:25	18.51 ± 0.020
Frying cycle 6	1:25	19.15 ± 0.010
WCO collected from hotel 80 frying cycle	1:25	19.37 ± 0.020
WCO collected from restaurant 95 frying cycle	1:25	19.70 ± 0.040
**Different ratio of Endod and the same amount of oil in the same frying cycle (b)**		
Frying cycle 6	0:100	22.52 ± 0.040
Frying cycle 6	1:50	20.17 ± 0.030
Frying cycle 6	1:33	19.46 ± 0.010
Frying cycle 6	1:25	19.15 ± 0.010
Frying cycle 6	1:20	17.96 ± 0.010
Frying cycle 6	1:17	16.56 ± 0.020

**Table 5:** The experimental results EIM of the prepared laundry soap.

Soap products prepared from	The blended ratio of Endod and oil (W/W, E/O)	% EIM
**Constant ratio of Endod and oil in different frying cycles (a)**		
Not-frying/fresh	0:100	0.104 ± 0.002
Frying cycle 1	1:25	0.387 ± 0.001
Frying cycle 2	1:25	0.606 ± 0.004
Frying cycle 3	1:25	0.942 ± 0.008
Frying cycle 4	1:25	1.070 ± 0.001
Frying cycle 5	1:25	1.132 ± 0.028
Frying cycle 6	1:25	1.31 ± 0.0500
WCO collected from hotel 80 frying cycle	1:25	1.45 ± 0.0500
WCO collected from restaurant 95 frying cycle	1:25	1.488 ± 0.008
**Different ratio of Endod and the same amount of oil in the same frying cycle (b)**		
Frying cycle 6	0:100	0.23 ± 0.028
Frying cycle 6	1:50	0.41 ± 0.010
Frying cycle 6	1:33	0.88 ± 0.040
Frying cycle 6	1:25	1.31 ± 0.050
Frying cycle 6	1:20	2.13 ± 0.030
Frying cycle 6	1:17	3.05 ± 0.030

**Table 6:** The experimental results TFM of the prepared laundry soap.

Soap products prepared from	The blended ratio of Endod and oil (W/W)	%TFM
**Constant ratio of Endod and oil in different frying cycles (a)**		
Not-frying/fresh	0:100	74.270 ± 0.200
Frying cycle 1	1:25	75.460 ± 0.200
Frying cycle 2	1:25	73.240 ± 0.100
Frying cycle 3	1:25	71.820 ± 0.180
Frying cycle 4	1:25	69.940 ± 0.020
Frying cycle 5	1:25	69.600 ± 0.100
Frying cycle 6	1:25	68.060 ± 0.110
WCO collected from hotel 80 frying cycle	1:25	66.730 ± 0.120
WCO collected from restaurant 95 frying cycle	1:25	66.460 ± 0.140
**Different ratio of Endod and the same amount of oil in the same frying cycle (b)**		
Frying cycle 6	0:100	63.410 ± 0.010
Frying cycle 6	1:50	64.320 ± 0.020
Frying cycle 6	1:33	66.570 ± 0.030
Frying cycle 6	1:25	68.060 ± 0.110
Frying cycle 6	1:20	70.910 ± 1.900
Frying cycle 6	1:17	71.10 ± 1.600

**Table 7:** The experimental results pH value of the prepared laundry soaps.

Soap products prepared from	The blended ratio of Endod and oil (W/W)	pH values
**Constant ratio of Endod and oil in different frying cycles (a)**		
Not-frying/fresh	0:100	9.77 ± 0.010
Frying cycle 1	1:25	9.69 ± 0.010
Frying cycle 2	1:25	9.45 ± 0.020
Frying cycle 3	1:25	9.26 ± 0.030
Frying cycle 4	1:25	9.82 ± 0.050
Frying cycle 5	1:25	9.42 ± 0.040
Frying cycle 6	1:25	9.44 ± 0.050
WCO collected from hotel 80 frying cycle	1:25	9.52 ± 0.010
WCO collected from restaurant 95 frying cycle	1:25	9.56 ± 0.030
**Different ratios of Endod and the same amount of oil in the same frying cycle (b)**		
Frying cycle 6	0:100	9.52 ± 0.020
Frying cycle 6	1:50	9.49 ± 0.010
Frying cycle 6	1:33	9.46 ± 0.040
Frying cycle 6	1:25	9.44 ± 0.030
Frying cycle 6	1:20	9.35 ± 0.050
Frying cycle 6	1:17	9.22 ± 0.020

**Table 8:** Cleaning power, foam formation, and stability of the prepared laundry soaps.

Soap products prepared from	The blended ratio of Endod and oil (W/W)	Foam		Cleansing power	Foam removed by water
		Height (cm)	Stability		
**Constant ratio of Endod and oil in different frying cycles (a)**					
Not-frying/fresh	0:100	7.00	Very high	Very high	Moderately
Frying cycle 1	1:25	8.10	Very high	Very high	Easily
Frying cycle 2	1:25	7.30	Very high	Very high	Easily
Frying cycle 3	1:25	6.90	Very high	Very high	Easily
Frying cycle 4	1:25	6.50	Very high	Very high	Easily
Frying cycle 5	1:25	6.00	Very high	Very high	Easily
Frying cycle 6	1:25	5.60	Very high	Very high	Easily
WCO collected from hotel 80 frying cycle	1:25	4.51	High	High	Easily
WCO collected from restaurant 95 frying cycle	1:25	4.43	High	High	Easily
**Different ratios of Endod and the same amount of oil in the same frying cycle (b)**					
Frying cycle 6	0:100	3.30	Good	Good	Slowly
Frying cycle 6	1:50	4.70	High	High	Moderately
Frying cycle 6	1:33	5.10	High	High	Easily
Frying cycle 6	1:25	5.60	Very high	Very high	Easily
Frying cycle 6	1:20	5.90	Very high	Very high	Easily
Frying cycle 6	1:17	6.40	Very high	Very high	Easily

In all soaps, the lye (NaOH) to water ratio (1:2.33, W/W) was added. The Physicochemical Properties of the Prepared Soaps were determined as follows.

### 3.3 Physicochemical properties of the prepared soaps

#### 3.3.1 Free caustic alkali

Free caustic alkali requires the roughness of any soap. The free caustic alkali (FCA) is the amount of alkali-free to counter and avert the soap from becoming oily. The FCA of the prepared soaps were determined and tabulated in Table 2. It was found that the FCA of the prepared soaps is 0.00% (Table 2). Thus, the free caustic alkali levels of the prepared soaps fall in the range for a good laundry soap since Excess free caustic alkali causes skin itching.

#### 3.3.2 Chloride content

The result shows the amount of chloride in all soaps was the same (0.00%) (Table 3). It is worth mentioning that Endod is used for hardening the soap instead of NaCl or KCl. Thus the product is totally chloride free and less susceptible to fracture under optimum conditions. Moreover, the results are less than some published literature (0.12%) [9], and the limit set by ISO 457 (1.5%) [36].

#### 3.3.3 Moisture and volatile matter content

The moisture content (MC) laundry soaps prepared from WCOs obtained from frying 1 up to 6 and collected from a hotel, and a restaurant, and blended with Endod had values 16.41 ± 0.050, 17.17 ± 0.050, 17.54 ± 0.010, 18.15 ± 0.010, 18.51 ± 0.020, 19.15 ± 0.010, 19.37 ± 0.020, 19.70 ± 0.040% respectively. The results clearly showed that the soaps made from blending WCOs and Endod had lower MCs than soap made from non-frying (20.07 ± 0.020%) without Endod. With the use of Endod, oil, lye (NaOH), and distilled water in a consistent ratio, the MCs of the soaps increased when the frying cycle increased (Table 4a). This is because the frequent frying had increased the acid value, causing the fatty acids in the oil to become more saturated [64], and resulted in a higher moisture content affecting the color of the oil, which changed from brown to dark brown, due to the presence of water that resulted from hydrolysis and aided the degradation of the oil [65].

In this study, the advantage of Endod to control the MC of a soap resulted due to the aforementioned reason. It absorbs excess water generated as a result of overheating making WCO a more suitable raw material for the preparation of a high-grade laundry soap (Table 4b. Despite this, all results show that the MC values of the prepared laundry soaps are below the EAS-recommended levels (30%), CES (26%), and for various countries, including Malaysia (36%), and the Philippines (30%) [34]. This indicates that the materials and procedures used in this investigation are very suitable to increase the shelf life of the product.

#### 3.3.4 Contents of ethanol-insoluble matter

The ethanol-insoluble matter (EIM) values of the prepared laundry soaps were observed to be in the range of 0.104 ± 0.002% to 3.05 ± 0.030%. The result showed that the soap prepared from WCOs and Endod had a greater EIM than soap made from fresh oil (0.104 ± 0.002%) without Endod. This is also another indication that by employing Endod, oil, lye (NaOH), and distilled water in a uniform ratio, the amount of EIM present in the soap increased as the frying cycle or the oil’s temperature increased (Table 5a). The effects of Endod in various ratios with the same amounts of oil, lye (NaOH), and distilled water were also examined in this study. In this instance, the EIM of the soaps grew along with the amount of Endod (Table 5b). The EIM values for blended WCOs and Endod soaps are very low compared to the value for the neem oil soap reported (19%) [5]. This is another indication that the WCOs and Endod soaps are of higher quality than the neem oil soap. The results also reveal that all EIM values of the made laundry soaps are below the EAS-recommended acceptable standard of (2.50%), CES-42-2017 (2.50%) [36], and for many countries, including British (2.00%), Malaysia (2.50%), and Philippines (5.00%) [34]. Nevertheless, laundry bar soap prepared from the fresh oil has a very high purity, soaps blended by blending WCOs and Endod have a high purity level upto the prescribed Endod to oil ratio (1:20; W/W) while the rest (1:16.67; W/W) have moderate purity.

#### 3.3.5 The total fatty matter content

The total fatty matter (TFM) content of soap samples involved in this investigation ranged from 63.41 ± 0.010 up to 75.46 ± 0.200%. The TFM values decreased from 75.46 ± 0.200 to 66.46 ± 0.140% as the frying cycle increased (Table 6a). This is because frequent frying has increased the acid value, which has led to more saturated fatty acids in the oil [64], and observed higher moisture content [65].

The effects of Endod in different ratios were determined. In the beginning, the impact of Endod was tested using non-frying oil without Endod or Endod to oil ratio (0:100) as the standard reference and frying 1 oil with Endod ratio Endod to oil (4:100). The results are 74.270 ± 0.200% and 75.460 ± 0.200% respectively (Table 6a). Similarly, the effects of Endod on the TFM of soaps were investigated by the WCO frying 6 without Endod ratio (0:100) and with Endod separately. In this case, the TFM of the soaps increased from 64.320 ± 0.020 to 71.100 ± 1.600% as the amount of Endod increased (Table 6b). This might be the result of Endod’s oil content and fatty acid composition as indicated by the previous studies [29]. Previous studies indicated that the active ingredients in the fruits of Endod are known for their saponin contents with oleanolic acid which when dried, powdered, and placed in water yields a foaming detergent solution having more powerful detergent properties [27]. Thus the natural saponin content of Endod contributed to the increase in the TFM value of the prepared soap. The TFM values of all prepared soaps exceeded those of the International Standard Organization (ISO), including the EAS and CES recommended level of (62%) [33], and for a variety of nations, namely Malaysia (60%), and the British (62%) (Table 6) [34]. Therefore, it is advised that all laundry bar soaps made from WCOs and Endod have higher cleansing power, cause less skin damage, and are grade-1/genuine soaps.

#### 3.3.6 The pH value

The pH measures the acidity or alkalinity of a substance. The pH results of the prepared soaps ranged from 9.22 ± 0.020 to 9.77 ± 0.010 (Table 7). This demonstrates that the results were significantly diverse when comparing the samples prepared from fresh and fried oil. Similarly, there are noticeable differences among soaps made with and without Endod. The pH range established by standard (9–11) [60] for acceptable laundry bar soaps encompasses all of the products’ pH values. Therefore, all samples satisfied the standard pH requirement for healthy soaps [35].

#### 3.3.7 Test of cleaning power, and foam formation and stability of the prepared laundry soap

**Fig. 4:**
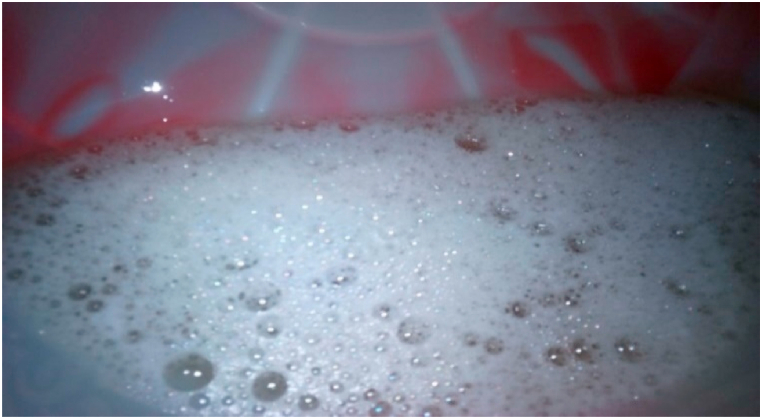
Endod berries powder high-foaming detergent solution.

Cleansing power, bubble, hardness, conditioning, creaminess, and lather formation are the principal traits generally used to assess first-class laundry soaps [9]. The detergency of soaps was tested on white, extremely dirty clothes that had been dumped on the road and heavily soiled with oil. As a result, it was found that the Cleansing power of soap derived from WCO and Endod was relatively better than without Endod. Similarly, soaps made from non-fried with Endod have a higher cleansing power than soaps made from WCO without Endod. This suggests that as the number of frying times increases, the soap’s detergency decreases. This is likely due to frequent frying increasing acidity, resulting in more saturated fat in the oil [64] and higher moisture content [65]. The higher the foam height, the better the soap lathers [6]. According to Hassan and Wawata, I.G foam height (4.6 cm) and above have the viable potential for utilization in soap and cosmetic industries [68]. Another study by Pak. J. Anal et al. indicated that foam height exceeding 2.3 cm could pass the lather test [67]. The results of this study showed that the soap made from frying cycle 1 with an Endod ratio of 1:25 has the highest foam height (8.1 cm). On the other hand, soap made from WCO produced a moderate height (3.3 cm). Other samples had a range of foam heights (from 4.0 to 8.1 cm) (Table 8). Increasing the concentration of Endod also increased the foam height. This is attributed to the oil and fatty acid content of Endod, which contains palmitic acid [29] and oleanolic acid [70], and the active ingredients in the fruit of the Endod plant which are known to have saponin (Fig. 4) [27].

The comparative physicochemical characterization result of the present work is summarized and given in Table S1. From the table, it can be boldly seen that some of the physicochemical characterization results of the current work are by far better than many reported works in similar areas. The improvement in the present work is attributed to the synergistic effect of the WCO and the Endod powder. The Endod berries power used as the raw material helps to reduce Cl ion content induced by the use of chloride salts alkali metal in the conventional soap preparation. Moreover, the plant contains saponins by nature and improves many of the physicochemical properties of the prepared soap.

## 4 Conclusion

The effect of blending endod with WCO was carefully investigated. Laundry soaps were prepared by using the proper amount of WCO and Endod powder in the laboratory for the first time. Physicochemical characterizations of the prepared soaps revealed that the products fit into the grade-1/genuine category of laundry bar soaps. Moreover, the result indicated that frying the oil has no significant effect on the quality of oil used as a raw material in soap making. The physicochemical characterization results of the soaps have also shown that all test parameters were within the recommended international standard limits, except for the soap prepared with an Endod to oil ratio of 6:100 (W/W), which exceeded EIM limits set by the EAS, CES (2.5%), and British (2%). Moreover, the result also revealed that a better soap could be prepared by mixing Endod berry powder with WCOs in the appropriate ratio range (2:100–5:100; W/W) of Endod to oil. Soap made by blending WCO and Endod has a comparable quality without Endod and non-fried made soap. Therefore, using WCO and Endod (*Phytolacca dodecandra*) for soap preparation has three advantages. First, it minimizes health problems and environmental pollution induced by direct using and discarding WCOs to the environment. Secondly, providing a new, cheap, and effective raw material (Endod plant which grows rapidly and bears fruit twice a year under the right climatic conditions) and is used for soap making instead. Above, all the use of such cheap active material also reduces the number of fillers required in soap production. The third is the blending of WCO and Endod replacing fresh palm oil, animal fats, and alkali metal salts (like NaCl and KCl) in soap making, reducing associated costs to soap production.

## Author contribution statement

Bahabelom Haile Abera, Tamene Tadesse Beyene: Conceived and designed the experiments; Performed the experiments; Analyzed and interpreted data; Contributed Reagents, Materials, Analysis Tools or Data; Wrote the paper.

Abebe Diro: Analyzed and interpreted data.

## Data availability statement

No data was used for the research described in the article

## Declaration of competing interest

The authors whose names are listed immediately below title of the manuscript certify that they have NO any known conflict interest with or involvement in any organization or entity with any financial interest, or non-financial interest (such as personal or professional relationships, affiliations, knowledge or beliefs) in the subject matter or materials discussed in this manuscript.

## Appendix A Supplementary data

Behabalom-WCO-SI _spl_1_spl_ Behabalom-WCO-SI _spl_1_spl_
