# Supplementary material for: The synergistic effect of waste cooking oil and endod (Phytolacca dodecandra) on the production of high-grade laundry soap
Source: Heliyon. 2023 Jun 1;9(6):e16889. doi: 10.1016/j.heliyon.2023.e16889 (PMC10279813; doi:10.1016/j.heliyon.2023.e16889)
Supplement: Behabalom-WCO-SI _spl_1_spl_ [file mmc1.docx]

**The Synergistic Effect of Waste Cooking Oil and Endod (*Phytolacca dodecandra*) on the Production of High-grade Laundry Soap**

**Bahabelom Haile Abera ^1^, Abebe Diro^1^, Tamene Tadesse Beyene ^1,^**^*^

*^1^Department of Chemistry, College of Natural Sciences, Jimma University, P.O. Box 378, Jimma-Oromia-Ethiopia,*

**Corresponding Author:*

*E-mail* [*tamene.tadesse@ju.edu.et*](mailto:tamene.tadesse@ju.edu.et)

Scheme S1. The saponification processes for soap preparation


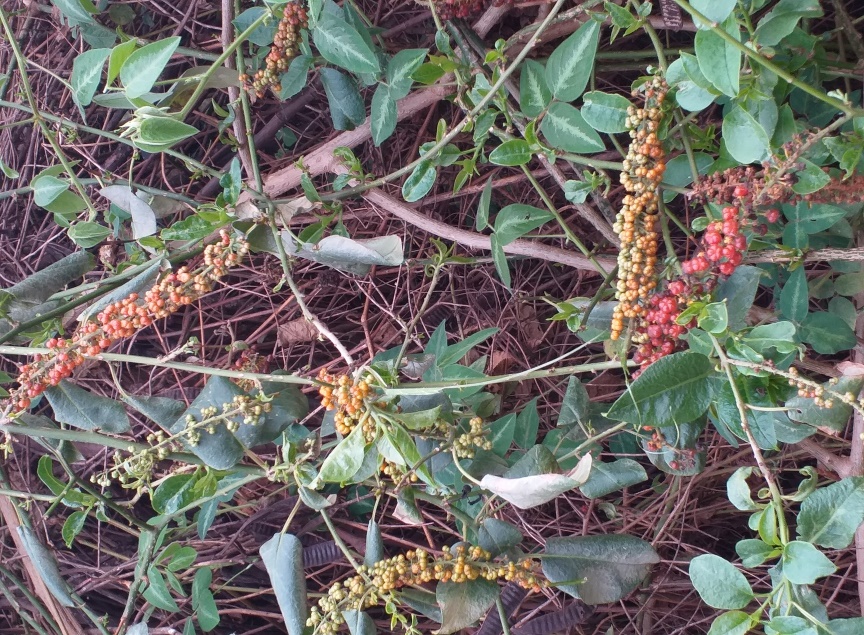


**Fig. S1.** Endod (Phytolacca dodecandra) plant


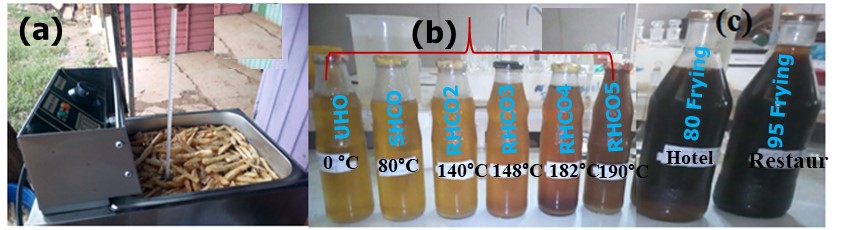


Fig. S2. Sample collection of WCOs (a) The stainless steel pan machine used for frying potatoes chips (b) RHCO samples for each of the six frying sessions (c) WCO samples collected from Hotel and restaurants frying sessions 80 and 95 respectively

**Fig. S3**. The pre-treatment of WCOs (a) Fresh ginger (b) Fresh pieces of Peeled ginger and dry at room temperature (c) The Peeled ginger pieces and WCOs cooked together for 30 min. at 90 °C (d) suction filtration (e) Treated WCOs from frying cycle 1-6 f) Treated WCOs collected from hotels and restaurants frying cycle 80 and 95 respectively


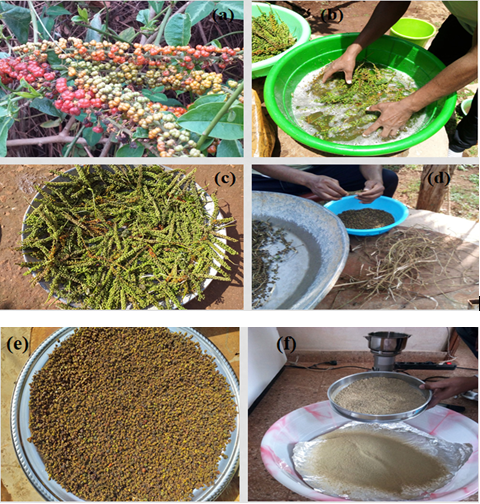


**Fig. S4.** Preparation of Endod berries powder particles size below 90 μm for making bar soap (a) Endod plant (b) remove dirty particles (c) drying (d) berries and stems separated (e) berries prepared f) berries crushed to powder using a grinder machine and filtered using a 90 μm sieve


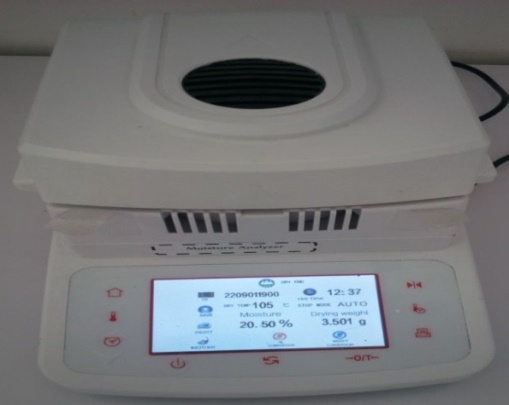


Fig. S5. A moisture analyzer that used to determine the moisture content of the samples

| **Study** | **Physicochemical Properties** | | | | | | | | | Reference |
| --- | --- | --- | --- | --- | --- | --- | --- | --- | --- | --- |
|  | **FCA**  **(%)** | **Cl^-^ content**  **(%)** | **Moisture content (%)** | **Insoluble matter (%)** | **TFM**  (%) | **pH** | **Foam** | | **Cleansing power** |  |
|  |  |  |  |  |  |  | **Height (cm)** | **Formation** |  |  |
|  | 0.01-0.06 | - | 1.38-8.44 | 55.46-89.0 | 11.72-49.65 | 9.79-10.02 | 0.50-3.50 | - | - | [3] |
|  | - | - | 11-21 | - | 40-86 | 8-10.5 | 1-9 | High | High | [6] |
|  | - | - | 2.45-10.94 | - | 88.89-96.24 | 9.53-9.96 | - | - | - | [8] |
|  | 0.19-0.22 | 0.12-0.20 | 6.67-14.47 | - | 75.42-88.53 | 9.86-10.56 | - | Medium | Good- High | [9] |
|  | 0.05 | 1.25 | 30 | 2.0 | 62 | - | - | - | - | [34] |
|  | 0.06-0.22 | - | 8.36-18.72 | 36-77 | 15.30-42.28 | 9.64-10.51 | - | Medium | very good | [35] |
|  | 0.2 | 1.5 | 30 | 2.5 | 62 | - | - | - | High | [36] |
|  | - | - | 5.40-15.12 | - | 59-91 | 6.29-11.39 | 2.3-8.5 | - | - | [69] |
|  | 0.0 | 0.0 | 16.56- 20.17 | 0.387- 2.13 | 66.46- 75.46 | 9.22- 9.82 | 4.43-8.1 | High | High | ***This study*** |

**Table S1**: Comparative summaries of the Physicochemical Characteristics of reported pieces of literature and the present work.
